# Achieving Clinical Success in Root Coverage and Aesthetic Improvement by Using Subepithelial Connective Tissue Grafts: A Report of Two Cases

**DOI:** 10.7759/cureus.72358

**Published:** 2024-10-25

**Authors:** Mohanasatheesh S, Nilofer Fajana, Anitha Balaji, Dheraj S

**Affiliations:** 1 Periodontics, Sree Balaji Dental College and Hospital, Chennai, IND; 2 Periodontics and Implantology, Sree Balaji Dental College and Hospital, Chennai, IND

**Keywords:** class i and class ii gingival recession, esthetic restoration, periodontal plastic surgeries, root coverage, single incision, sub epithelial connective tissue graft

## Abstract

Over the years, several modalities have been established to treat gingival recession. Nonetheless, subepithelial connective tissue grafts (SECTGs) remain a dependable technique for root coverage, mainly due to their superior vascularization. However, the procurement of the CTG is susceptible to technique sensitivity; consequently, various methodologies have emerged to harvest CTGs to mitigate donor site trauma. In this article, we present two clinical cases of gingival recession addressed by the application of SECTGs. Both instances involved patients in good health with localized gingival recession measuring 6 mm and 7 mm in the mandibular incisors. The grafts were procured from the hard palate to rectify the recession areas. This report emphasizes the importance of minimizing trauma to the donor site, alleviating postoperative discomfort, and achieving complete root coverage at the recipient site with a favorable aesthetic result. Follow-up assessments at the first, fourth, and eighth weeks revealed the achievement of full root coverage and the patients reported satisfaction with reduced postoperative discomfort.

## Introduction

Gingival recession refers to the displacement of the soft tissue margin apical to the cementoenamel junction [1]. The concept of connective tissue graft (CTG) was first introduced by Alan Edel in 1974 for increasing the width of the gingiva. Since then, its applications have expanded greatly in dentistry [2]. In particular, the aesthetic aspect of gingival recession is a challenge for both the patient and the dental professional [3]. The marginal tissue in Miller's class II gingival recession extends beyond the mucogingival junction without loss of soft tissue or interdental bone. The dental roots are often exposed to this disease, increasing the risk of root caries, causing dentin sensitivity, and creating aesthetic issues [4]. Also, these issues need to be addressed not only to improve the patient’s oral health but also the overall quality of life and self-esteem. The subepithelial CTG (SECTG) is highly efficacious for attaining root coverage in cases of gingival recession [5]. The surgical technique involves harvesting connective tissue from the palate and grafting it to the site of recession to cover the exposed root and increase the thickness of gingival tissue [5]. The CTG method is particularly useful in the aesthetic area, where the achievement of a natural and harmonious outcome is key.

## Case presentation

Case 1

**Figure 1:**
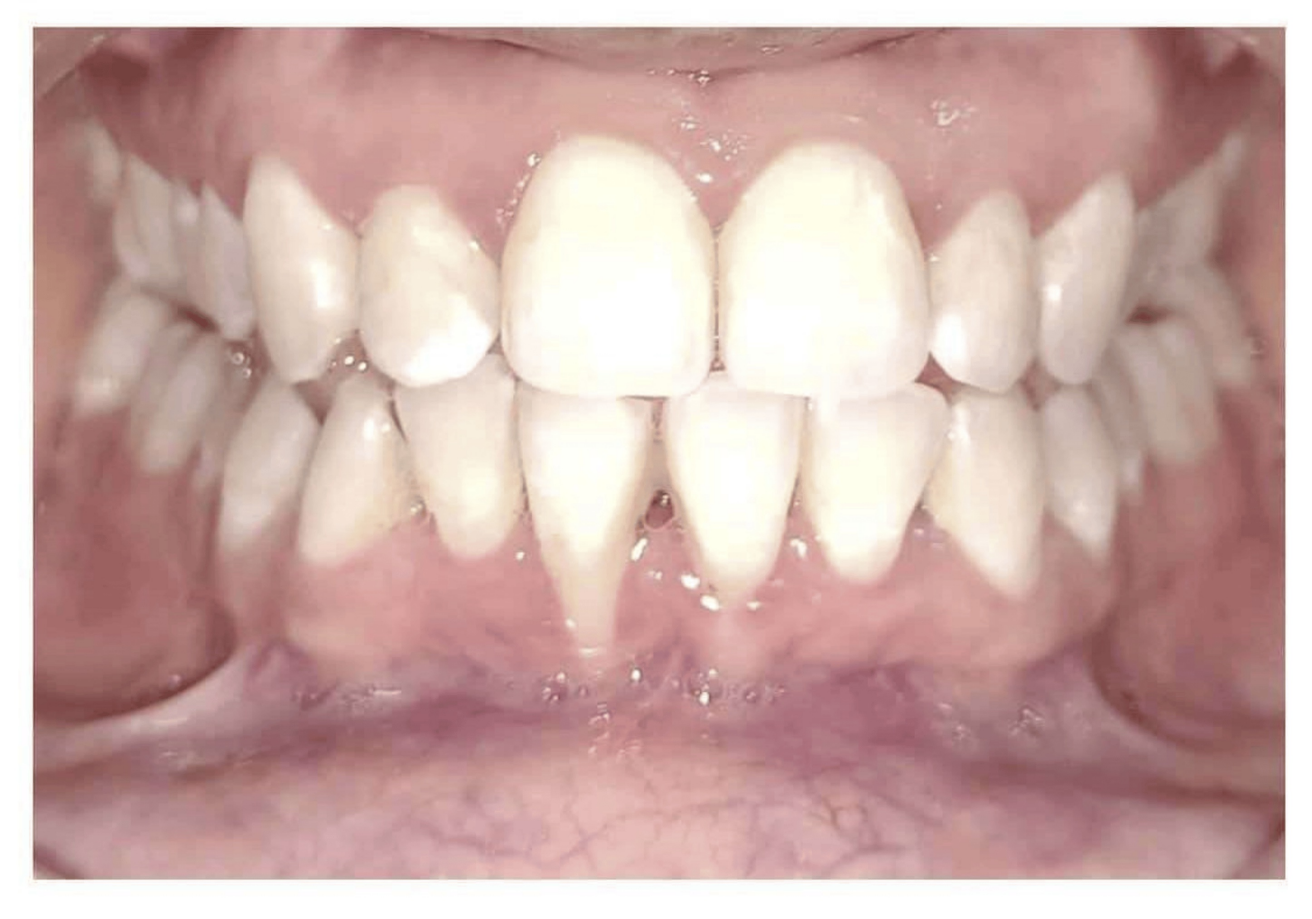
Case 1 - preoperative clinical picture

A 23-year-old female patient presented with five months of persistent gingival recession in the lower anterior region. Her general health was found to be in good condition. An intraoral examination demonstrated that tooth 41 was associated with Miller's class III gingival recession (Figure 1). Tooth 41 measured 6 mm in length and 3 mm in width, had receded by a length of 6 mm, and was categorized as Miller's class II due to insufficient keratinized gingiva and a probing depth of 5 mm. Informed consent was obtained from the patient after a complete explanation of the suggested root coverage treatment plan.

Case 2

**Figure 2:**
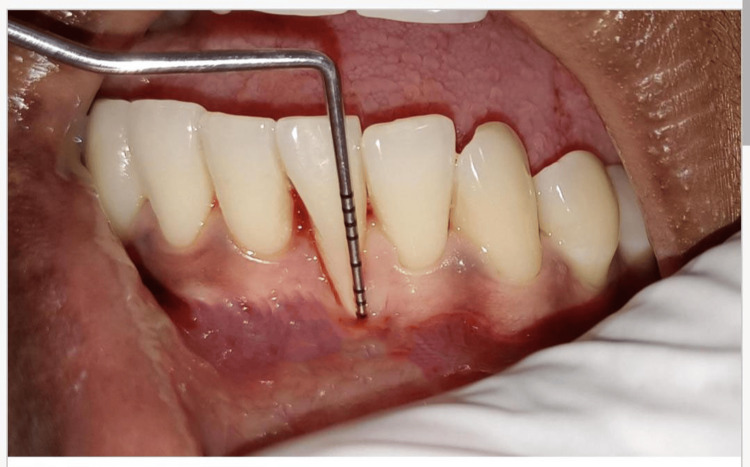
Case 2 - preoperative clinical picture

A 30-year-old female patient presented with gingival recession in the lower front teeth for the past eight months, with no underlying health conditions. Miller's class III gingival recession at mandibular incisor 31 was detected during an intraoral examination (Figure 2). It measured 8 mm in length and 3 mm in width, had a 7 mm probing depth, and had insufficient keratinized gingiva. Following a thorough consultation, informed consent was obtained from the patient before developing a root coverage treatment plan.

Surgical procedure

**Figure 3:**
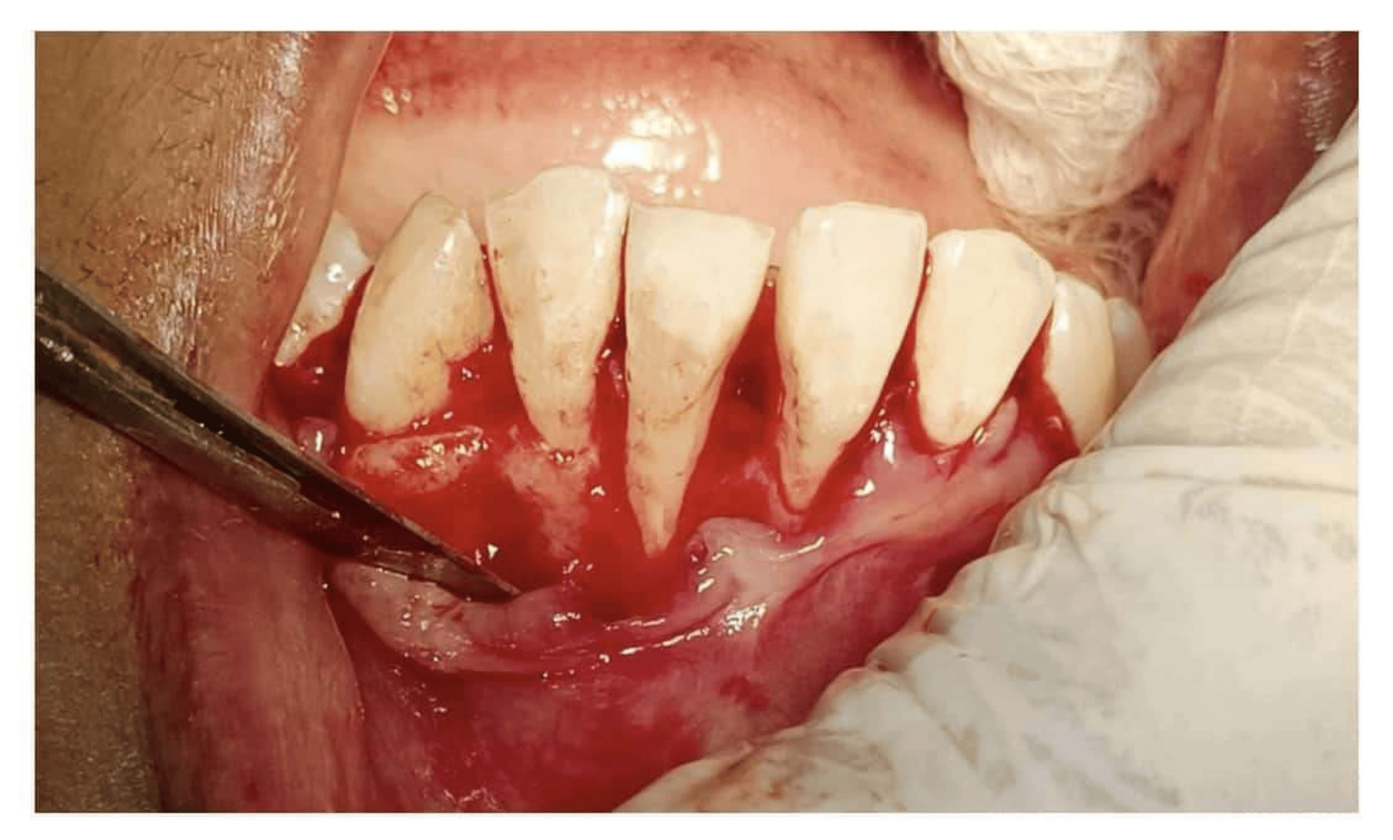
Case 1 - full-thickness flap elevated

**Figure 4:**
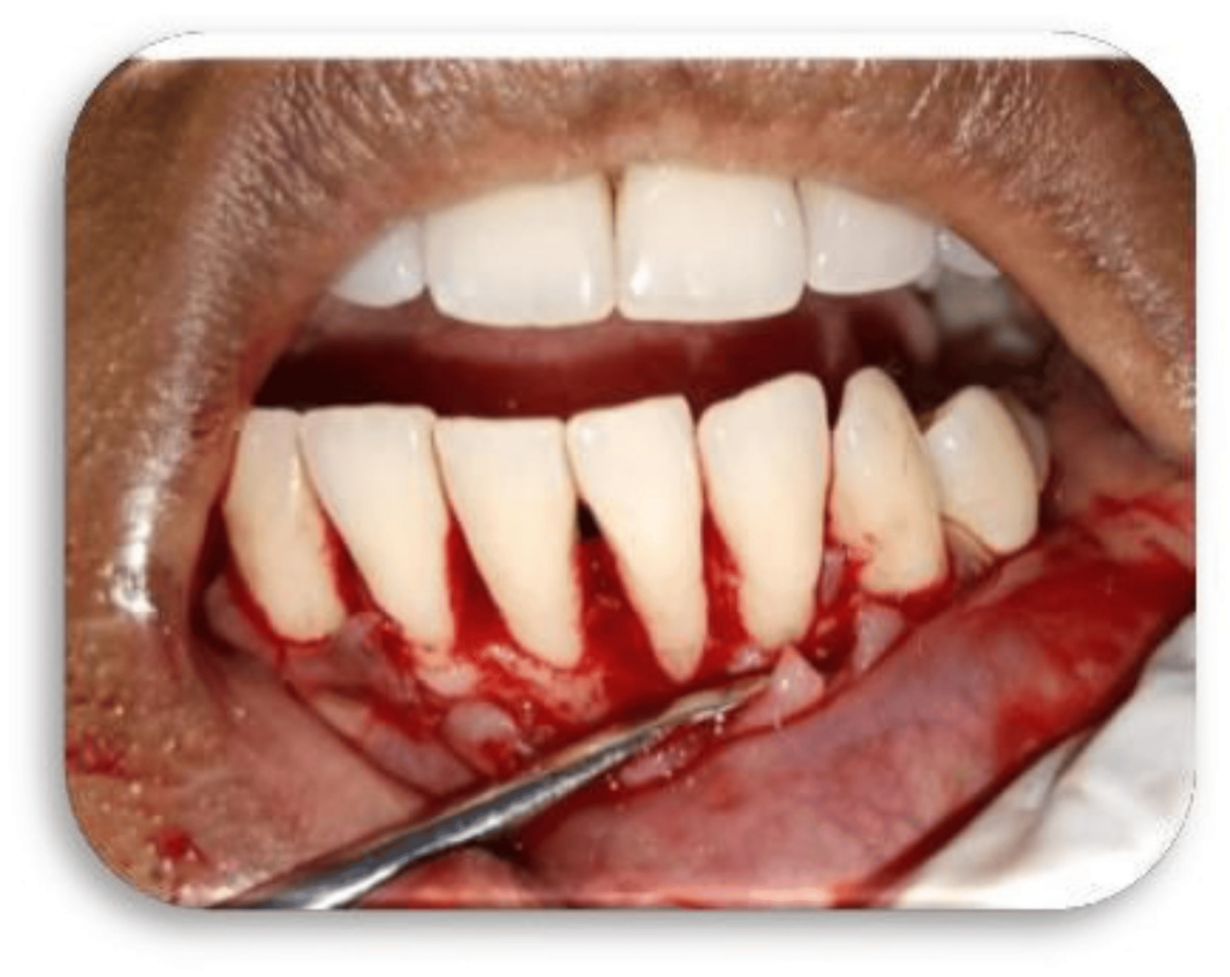
Case 2 - full-thickness flap elevated

The patient was taught efficient plaque control techniques. Full-mouth scaling as well as root planning was performed under local anesthesia (2% lidocaine with 1:100,000 epinephrine), adhering to aseptic standards. The recipient site had been prepared by raising a full-thickness flap on the labial surface of teeth 31 and 41 respectively, extending to adjacent teeth and surpassing the mucogingival junction to enable proper coronal repositioning (Figures 3-4).

**Figure 5:**
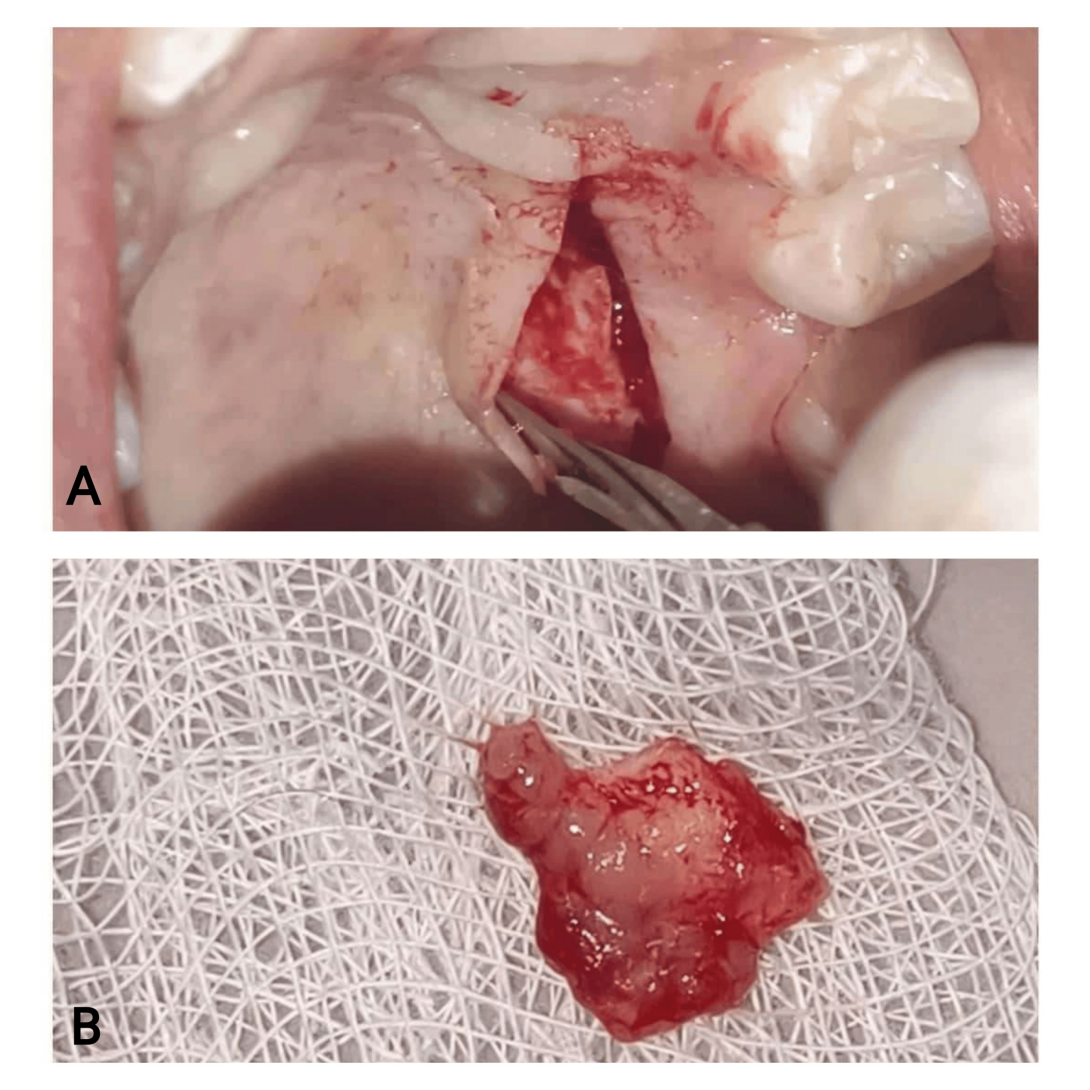
Case 1 - connective tissue harvest 5A: connective tissue harvesting using a single incision. 5B: 1.5-mm-thick connective tissue harvested

**Figure 6:**
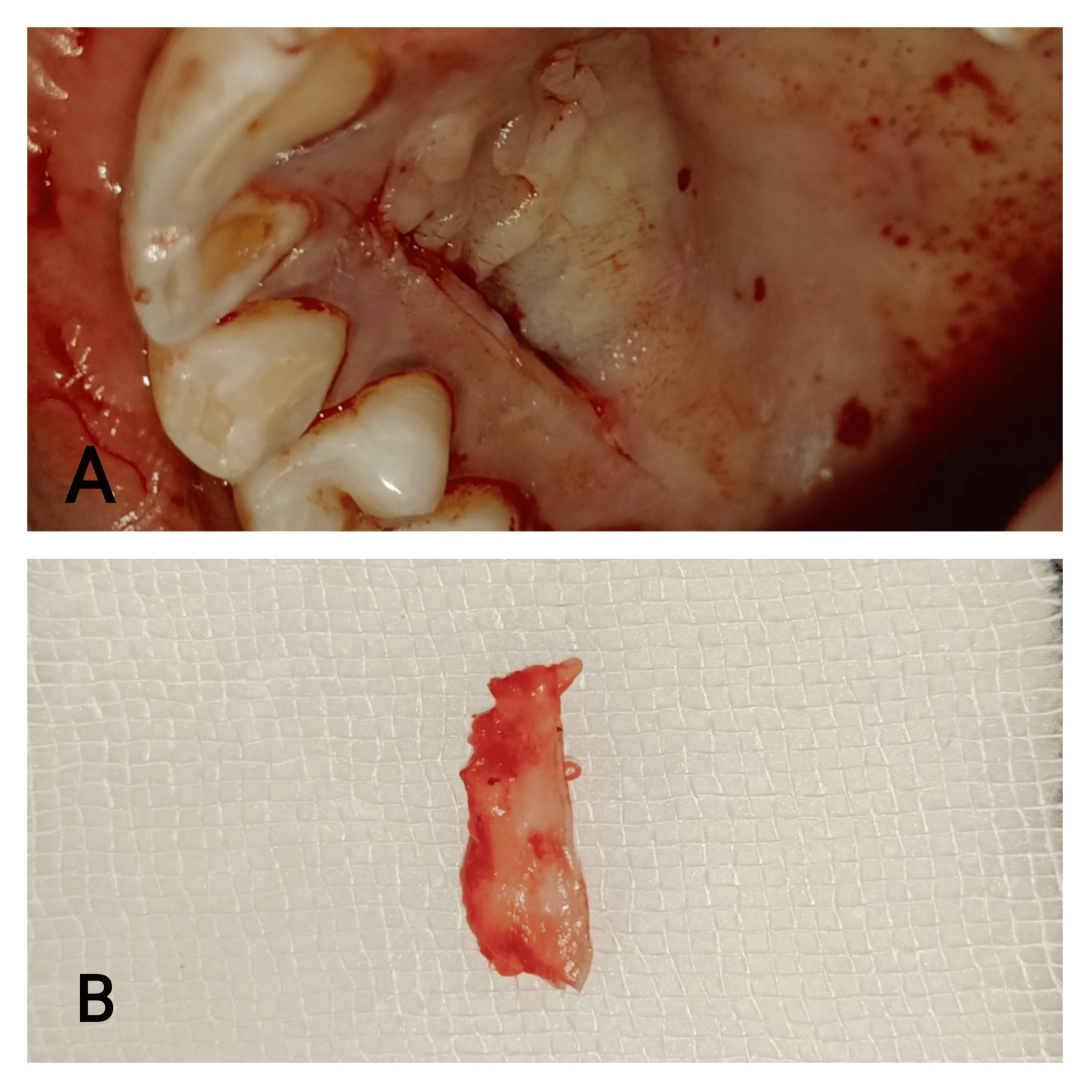
Case 2 - connective tissue harvest 6A: connective tissue harvesting using a single incision. 6B: 1.5-mm-thick connective tissue harvested

By employing the single incision method developed by Hurzeler and Weng, the connective tissue was extracted from the palate. A horizontal incision was executed 2 mm from the marginal gingiva, with the blade positioned to undermine the flap (Figures 5A, 6A). A 1.5-mm-thick SECTG was harvested from the hard palate of the first molar to the distal canine region (Figures 5B, 6B).

**Figure 7:**
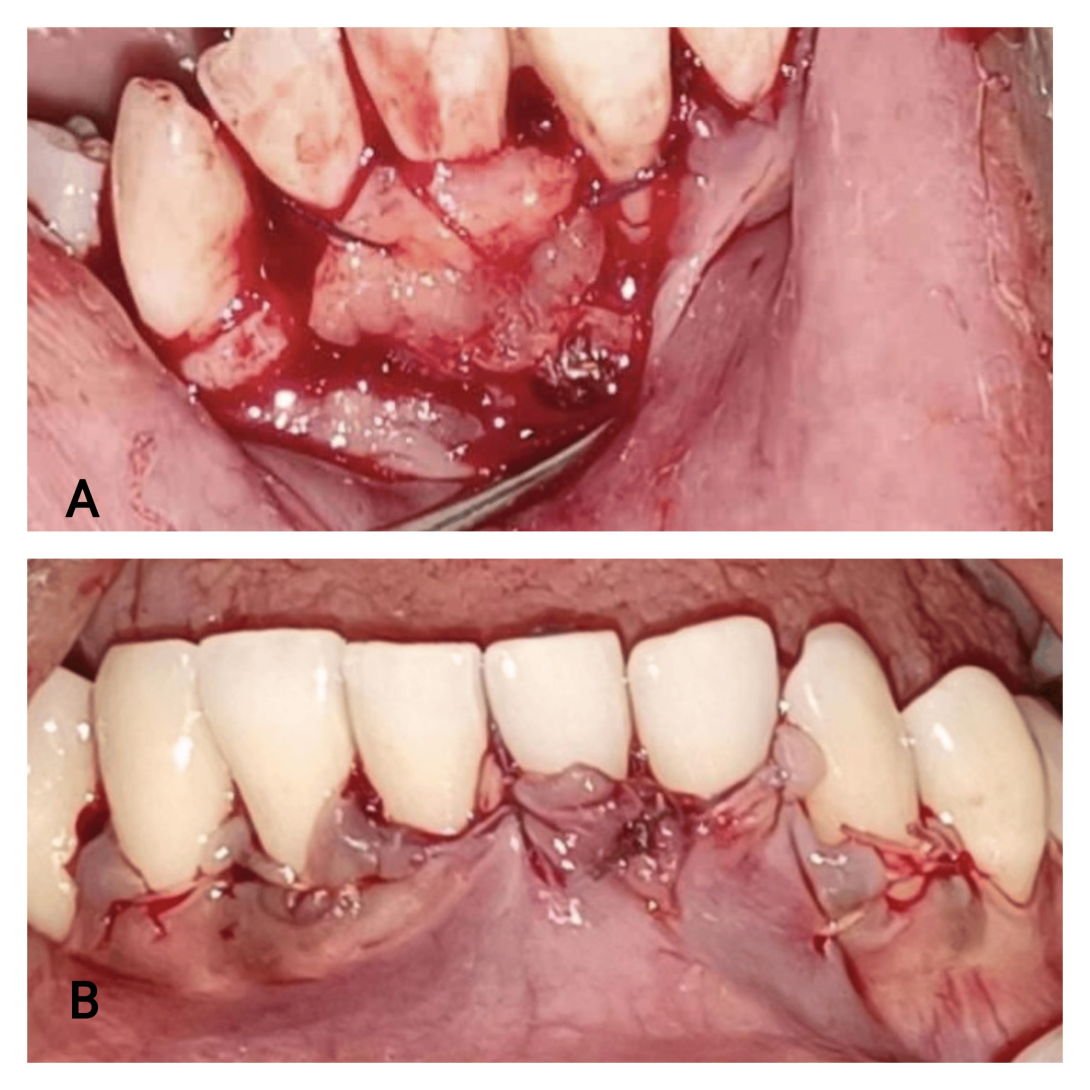
Case 1 - connective tissue stabilization and suturing 7A: connective tissue stabilized with silk suture. 7B: flap approximated

**Figure 8:**
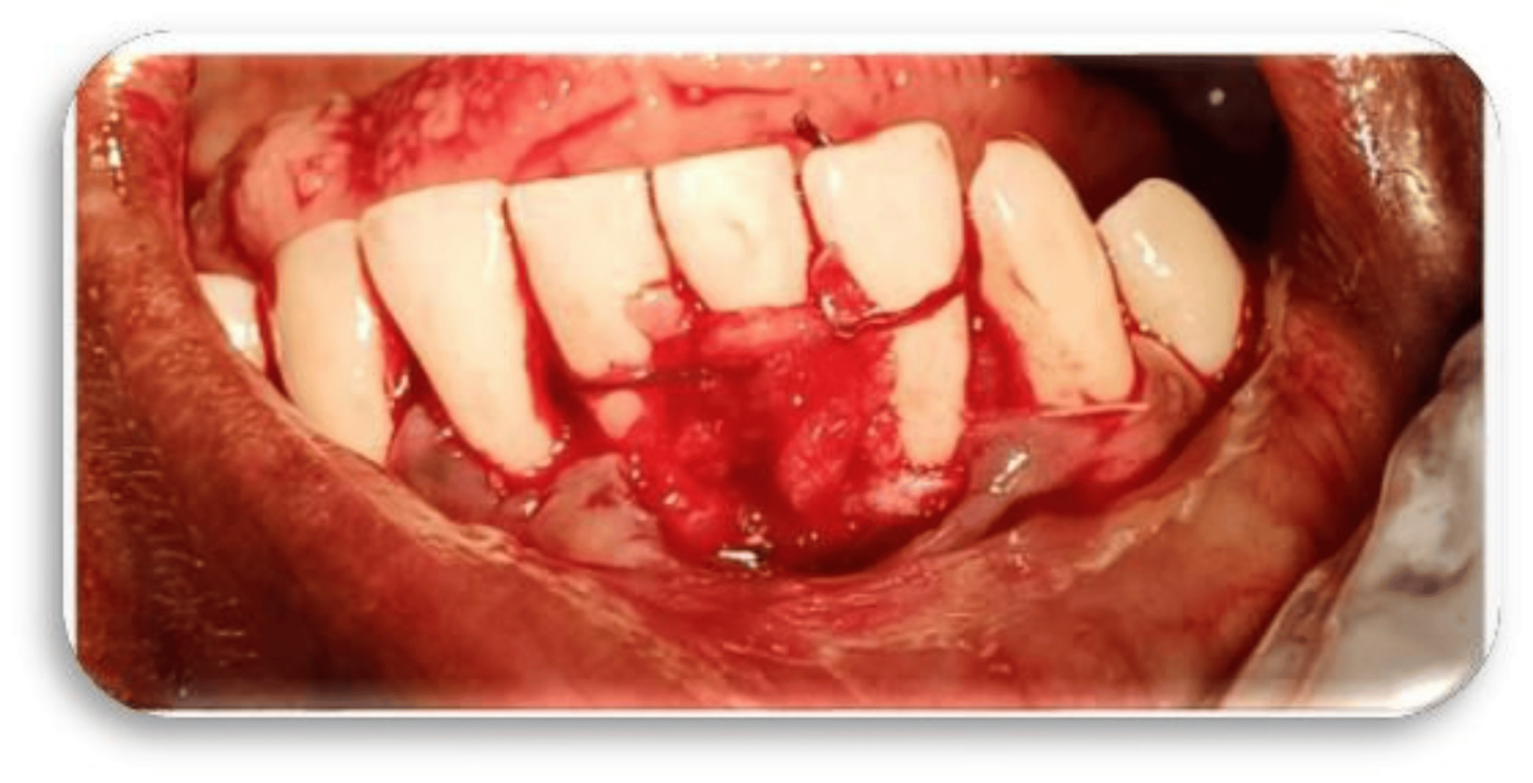
Case 2 - connective tissue stabilization

The graft was affixed to the recipient site by utilizing non-resorbable 4-0 black silk surgical sutures (Figures 7A, 8); the flap was approximated with 4-0 silk suture by ensuring proper stabilization and coverage of the connective tissue, and the donor site was also sutured (Figure 7B).

**Figure 9:**
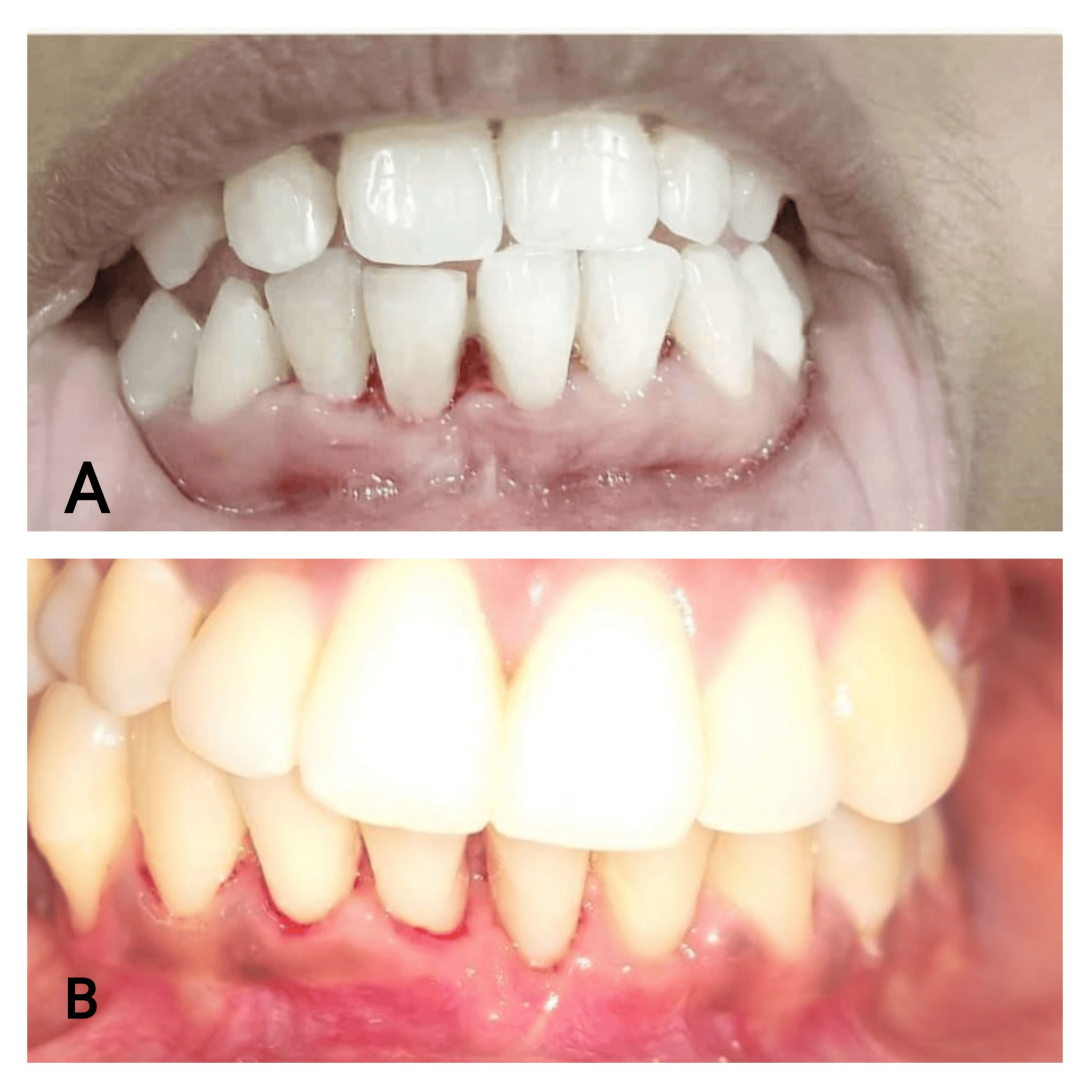
Postoperative clinical picture 9A: case 1. 9b: case 2

Following surgery, postoperative care instructions were given, and patients were advised to take NSAIDs and antibiotics twice a day for a week, along with a mouthwash containing 0.2% chlorhexidine gluconate. They were also instructed to avoid brushing the surgical site for a week. Follow-up examinations at one, four, and eight weeks demonstrated a favorable outcome for root coverage, an augmentation in the width of the attached gingiva, and good healing (Figures 9A, 9B).

## Discussion

A 2010 Cochrane systematic review found that CTGs are more suitable for cases that need increased keratinized tissue and root coverage. Chambrone et al. and Chambrone and Tatakis found that SECTGs provide the greatest clinical outcomes with higher percentages of mean as well as total root coverage and considerable increases in keratinized tissue, further reinforcing this theory [6,7]. Periodontal plastic surgery aims to attain a pink appearance. Using an autogenous CTG is the optimal method for obtaining full root coverage with long-term stability, and it also helps to widen the associated gingiva [2]. The clinical difficulty of obtaining a sufficient graft with simple conventional methods has prompted researchers to explore new or modified techniques. The choice of approaches depends on tissue integrity, clinical expertise, anatomical position, and techniques involved [8]. A thorough review and meta-analysis of SECTGs revealed predictable outcomes with an increase in mean root coverage [8]. It is challenging to procure large volumes of grafts with minimally invasive techniques and no postoperative complications [9].

Techniques for harvesting CTGs can include vertical incisions, techniques without vertical incisions, double incision methods, single incision methods, methods involving an epithelium band, methods that leave the periosteum on bone, and de-epithelialized CTG techniques. Liu and Weisgold provided classifications for various incision designs [10]. The finest technique involves the one that is atraumatic, and simple to procure with minimal postoperative complications [11]. The technique by Hurzeler and Weng was implemented in two case reports for harvesting donor tissue, and it involves a single horizontal incision with a full-thickness flap that provides optimal vascularization of the cover flap, a smaller number of sutures, painless wound healing, grafts with various dimensions, good postoperative healing, and decreased patient morbidity. [12]. A Barraquer cataract knife and AVS blade were used in a modified single incision technique to achieve faster healing and less postoperative pain in recession management by Kumar et al. in 2013 [13].

Research has shown that having epithelium and sufficient blood supply is crucial for predictable results, which can be attained via proper access and control [5]. Each technique of subepithelial connective graft has its own advantages and disadvantages, depending on the objective of the procedure, anatomical limitations, postoperative complications, and clinical expertise [14].

## Conclusions

Connective tissue is optimal for soft-tissue augmentation in gingival recession treatment. The two cases we reported show the effectiveness of obtaining CTG through a single incision, highlighted by better patient outcomes and significantly reduced postoperative pain. This success has led to the expansion of their use in periodontal aesthetics. However, these methodologies require advanced proficiency and expertise. Consequently, practitioners need to be well-versed in tissue handling, graft harvesting, restrictions, and potential problems. In cases where aesthetics is the main consideration, SECTG is a dependable method to ensure root coverage in Miller's class II gingival recession. This case report adds to the evidence affirming the technique's effectiveness and emphasizes the importance of tailored treatment plans for superior clinical and aesthetic outcomes.
